# Impact of coverage and guest residue on polyproline II helix peptide antifouling

**DOI:** 10.1557/s43579-024-00674-w

**Published:** 2024-11-11

**Authors:** Rebecca S. Ahn, Henry T. Grome, Sogol Asaei, Geeta Verma, Christina S. Dang, Harihara Baskaran, Julie N. Renner

**Affiliations:** Department of Chemical and Biomolecular Engineering, Case Western Reserve University, Cleveland, USA

**Keywords:** Adsorption, Adhesion, Biomaterial, Surface chemistry, Coating

## Abstract

Polyproline II (PPII) peptide sequences are recognized as promising biomaterials because of their attractive antifouling properties. However, the mechanisms behind their antifouling behavior have not been fully characterized. In this work we show that PPII peptide coverage, controlled by adsorption time, significantly reduces the fouling of bovine serum albumin (BSA, a model foulant). In addition, guest residues introduced into the PPII sequence are shown to significantly impact BSA adsorption as well as human mesenchymal stem cell (hMSC) spreading. This research will help guide future PPII peptide designs for incorporation into novel biomaterials.

**Graphical abstract:**

**Figure:**
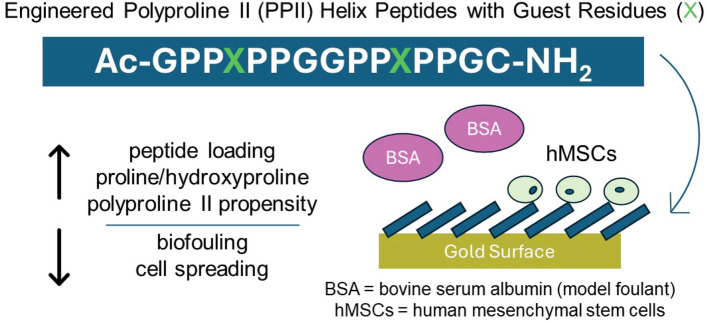
A guest residue system was utilized to understand properties that impact the ability of polyproline II helix peptide monolayers to resist the fouling of BSA and influence human mesenchymal cell spreading.

**Supplementary Information:**

The online version contains supplementary material available at 10.1557/s43579-024-00674-w.

## Introduction

According to the World Health Organization (WHO), non-communicable diseases such as cardiovascular diseases, cancer, and diabetes cause about 74% of all annual worldwide deaths.^[1]^ To treat these conditions, monitoring devices that interface with complex biological fluids and require specialized coatings are gaining attention.^[2,3]^ Antibiofouling technologies are critical to the operation of these implantable devices and can serve to aid in more specific and sensitive sensing.^[4,5]^

It has been observed previously that antifouling is crucially related to hydration.^[6]^ Among many technologies, poly(ethylene glycol) (PEG) is one of the most recognized antifouling materials. PEG effectively prevents the adsorption of undesired debris with its hydrated brush monolayer structures.^[7,8]^ However, some studies have raised concerns with the use of PEG because it can accumulate in the human body over time and produce an immune response.^[9,10]^ Zwitterionic polymers also have high antifouling properties due to their high hydration.^[11–14]^ However, these materials require a specific pH range (not highly acidic or basic) to maintain the zwitterionic nature, making these materials less versatile for certain parts of the body, and for sensing applications where sample pH can vary. While zwitterionic peptide sequences have been designed, other peptide sequences that do not rely on charge for their functionality would be desirable.

Polyproline II helix (PPII) peptides, which do not rely on charge for their functionality, have recently emerged as promising antifouling materials. PPII peptides first gained attention as effective anchors of other antifouling peptides.^[14–16]^ These studies implied that the rigid structure of the PPII anchor led to a high packing density of peptide connected to the PPII sequence.^[14,17]^ We as well as others demonstrated that PPII peptides have antifouling properties without the attachment of additional antifouling sequences.^[18,19]^ While it was previously shown that the length of the PPII peptide sequence is an important factor in antifouling,^[18]^ none of these studies have investigated the impact of guest residue content, which is knowledge critical to designing multi-functional antifouling surfaces.

This study explores the hypothesis that guest residue content impacts PPII peptide antifouling properties. This was done by using a guest residue-containing PPII sequence that was designed based on previous work in our lab which showed that the sequence prevented bovine serum albumin (BSA, a model foulant) adsorption when bound to gold.^[19]^ BSA is a common model protein due to its stability, availability, and low cost. It is used often in fouling experiments, including in work by others working with oligo-prolines.^[18]^ In the research reported here, we quantify peptide loading and measure the impact on fouling for PPII peptides of similar length, but varying amino acid content. We characterize the secondary structure of PPII peptides and the surface properties of the peptide-functionalized substrate. We also investigate the adhesion and spreading of human mesenchymal stem cells (hMSCs) seeded on PPII peptide-functionalized gold surfaces to understand the impact of the peptides on clinically relevant cells.^[20]^ An attractive property of hMSCs is that they are multipotent, which can be influenced by their shape and spreading on surfaces.^[21]^ Collectively, this research aims to develop insights that will guide the design of these peptides for use in future biomaterials.

## Materials and methods

### Chemicals and reagents

DNase/RNase-free water was purchased from Invitrogen. Deionized (DI) water (H_2_O) was generated through Western Reserve Water System (1–10 MΩ) and a Millipore system which produced DI water at 18 MΩ. Ammonium hydroxide solution (NH_4_OH, 30%) was purchased from Alfa Aesar. Hydrogen peroxide solution (H_2_O_2_, 25%) was purchased from Sigma-Aldrich. Sodium dodecyl sulfate (SDS, C_12_H_25_NaO_4_S, 99%) was purchased from Dot Scientific. Nitrogen gas (N_2_ > 99%) and ultrahigh-purity nitrogen gas (UHP N_2_, > 99.998%) were purchased from Airgas. The gold (Au)-coated quartz crystal (QSX 301) sensors for quartz crystal microbalance with dissipation (QCM-D) monitoring were from NanoScience. Peptides (purity > 95%) were purchased from GenScript. Bovine serum albumin (BSA) was purchased from Fisher.

### Peptide design

The peptides in this study were based on sequences developed by Brown et al.^[22]^ The base sequence for this work was Ac-GPPXPPGGPPXPPGC-NH_2_, where X represents a guest residue. Cysteine was included to facilitate binding to gold via a covalent thiol bond. For all peptides, the N-terminus was acetylated, and the C-terminus was amidated to improve stability.

### Circular dichroism (CD)

A circular dichroism (CD) spectrometer (Jasco J-815, Jasco, Tokyo, Japan) was used to analyze the secondary structure of the peptides in solution. Peptides were diluted with Millipore-filtered DI water to 100 mM peptide concentration. Before adding the sample, the cuvette was rinsed with Millipore-filtered water and dried. Millipore-filtered DI water served as a baseline for the sample. Each solution was read from 190 to 260 nm at room temperature and scanned at a speed of 50 nm/min in a quartz cuvette with a 0.1-cm path length. The measured spectra were baseline corrected and smoothed through the Spectra Manager ver. 2.0 program (Spectra Analysis processing tool) which is equipped with the spectrometer.

### Contact angle

Water contact angle measurements on peptide-functionalized and bare gold QCM-D sensors were taken using a Dataphysics OCA 15 plus contact angle measuring device (Wisconsin) with SCA 20 software. QCM-D sensors cleaned with piranha solution were immersed and incubated for 24 h in 10 mL of 10 ppm peptide solution in Millipore-filtered DI water. Two bare gold control samples were prepared with one that was not immersed in Millipore-filtered DI water, and one that was immersed in Millipore-filtered DI water for the same amount of time as the peptide-functionalized samples. Droplets of 5 µL Millipore-filtered DI water were pipetted over each QCM-D sensor. Each water droplet was equilibrated for 10 s before photographing. Photos and measurements were taken automatically using SCA 20 software. The average of left and right angles was determined for each droplet, and all reported contacted angle measurements were averages of three repeats (consisting of three separately prepared sensors).

### Quartz crystal microbalance with dissipation (QCM-D) analysis of adsorption

A quartz crystal microbalance with dissipation monitoring (QCM-D, NanoScience, Gothenburg, Sweden) was utilized to quantify the adsorption of peptide and foulant with time. The temperature of the flow module (QFM 401, NanoScience, Gothenburg, Sweden) was set to 18°C throughout the experiment. Initially, DNase/RNase-free water was pumped at a flow rate of 0.150 mL/min into the flow module until the frequency stabilized, defined by no more than 0.3 Hz frequency shift over a period of 10 min. After 10 min of stable baseline, 10 µg/mL peptide samples diluted with DNase/RNase-free water were pumped past the sensor at 0.150 mL/min for 1–24 h according to the needs of the experiment. After peptide functionalization, DNase/RNase-free water was pumped into the module at 0.150 mL/min for 10 min to remove any loosely bound peptide. A solution of 10 µg/mL bovine serum albumin (BSA) diluted with DNase/RNase-free water was used as foulant and pumped past the sensor for 110–120 min after peptide functionalization and rinsing. If dissipation did not increase more than 0.5 ppm per 10 Hz of frequency change (as was the case for peptide adsorption), a rigid film was assumed, and the Sauerbrey Equation ^[25]^ was used to calculate the mass. The 9th overtone was used to analyze the data. Since the shift in dissipation increased by more than 10% of frequency during BSA adsorption, we used a viscoelastic model to estimate BSA mass.^[26]^ The viscoelastic model used 3rd, 5th, 7th, 9th, and 11th overtones of the frequency and dissipation data. The module and sensors were cleaned as described in our prior work.^[19]^ If significant drift was observed in dissipation or frequency, the runs were terminated.

### Cell culture platform design

Gold-coated cell culture plates were made to study the adsorption and spreading of hMSCs on selected PPII peptides. The fabrication process was divided into two main steps: making the well divider and coating the surface with gold. Well dividers were made using polydimethylsiloxane (PDMS) (SYLGARD™ 184 Silicone Elastomer Kit, DOW). Briefly, the silicone elastomer base and silicone elastomer curing agent were mixed at a 10:1 mass ratio for 5 min before degassing under vacuum in a chamber to remove all air bubbles. For the easy release of the mold, the surface of a glass Petri dish (Corning^TM^ 70165-101, New York, USA) was treated with a thin layer of soap ethanol solution (10% soap solution mixed with 90% of ethanol), and the solution was evaporated in a 40°C oven. Then 45 g of the PDMS mixture was poured into the Petri dish. The mixture was baked in the oven for 45 min at 100°C. Once the baked PDMS was removed from the dish, holes were made by using a 10-mm biopsy punch (Acuderm Inc—P1050, Florida, USA). The outskirt of PDMS was trimmed to prevent undesirable bonding. The PDMS layer was rinsed with 70% ethanol in Western Reserve Water System DI water and dried with N_2_ gas. The PDMS layer and a glass cell culture dish were exposed to oxygen plasma (SPI Plasma Prep II, Pennsylvania, USA) for 60 s, after which they were bonded together in a 40°C oven overnight with the PDMS layer and the dish held tightly together by heavy aluminum blocks. The petri dish bonded to the PDMS layer was then rinsed with Western Reserve Water System DI water and 70% ethanol and dried with N_2_ gas. Fully dried dishes were autoclaved and underwent radio frequency (RF) plasma treatment for 60 s.

Once the well divider was prepared, the next step was coating surfaces with gold for peptide binding. Ti/Au films were deposited onto the plates with the dividing wells using a magnetron sputtering system (Denton Vacuum Discovery 18) with three DC cathodes. The two cathodes used contained three-inch diameter targets, 99.995% Ti and 99.99% Au. The cathodes were located at the top of the deposition chamber with the sample located on a rotating plate below. 5 nm of Ti was deposited for 22 s first for use as an interlayer between the base glass dishes and the outer Au layer to improve the adhesion of the Au layer. 50 nm of Au was deposited over 46 s. Both depositions were operated at 250 W DC at a base pressure of 7.7 × 10^–7^ torr and deposition pressure of 3–4 mtorr. After sputtering, dishes were autoclaved prior to cell culture.

### Cell antifouling analysis with hMSCs

Chemicals and reagents for growing and obtaining hMSCs from bone marrow are detailed in a previously published paper.^[27]^ Gold-coated cell culture plates were further modified with 10 µg/mL peptide solution using PPII_P, PPII_L, and a negative control solution with no peptide. The solutions were filtered by a 0.22-µm syringe filter prior to use. All the wells were functionalized for 3 h at 37 ℃. After the functionalization of the peptide, each well was rinsed with phosphate buffer saline (PBS) two times to remove any loosely bound peptide from the surface. Passage 1 (P1) human MSCs (hMSCs) were obtained from bone marrow donated by healthy volunteers as per an IRB-approved protocol by the Stem Cell Core Facility of the Case Comprehensive Cancer Center, Case Western Reserve University, OH. The hMSCs were isolated using previously published methods.^[28] ^The hMSCs were seeded into the peptide-functionalized gold-coated cell culture plates at a density of 50,000 cells in each well. After two hours of hMSC attachment in the incubator at 37 ℃, each well was placed under a cell culture microscope (Olympus CKX41 Inverted Microscope, Evident, Tokyo, Japan). The pictures of cell spreading samples were taken using a Galaxy S23+ (Samsung, Suwon, South Korea) camera application with the 4× zoom setting. A hemocytometer was imaged to calibrate the scale. ImageJ was used to analyze pictures by drawing an outline around each cell and to measure area and circularity.

### Statistical analysis

All statistical analysis was performed with Minitab Version 21.3.1. A significance level of *α* = 0.05 was utilized. Tukey’s *post hoc* test was applied to determine statistical differences between groups if single factor analysis of variance (ANOVA) showed a statistically significant impact of the factor being investigated. Simple linear regression was performed to analyze the relationships between parameters (e.g., mass of the peptide and the amount of BSA fouling).

## Results and discussion

### The impact of PPII guest residue on BSA fouling

Circular dichroism was used to analyze the secondary structure of PPII peptides with varying guest residues and confirm that PPII propensity varied according to the scale developed by Brown et al*.*^[22,29]^ Figure S1(a) shows the circular dichroism spectra for each peptide in Table I. Proteins and peptides with PPII secondary structures tend to have a large negative band around ~ 200 nm and a positive band around ~ 220 nm. A strong negative peak is observed around 200–210 nm for all peptides. A distinct positive peak is observed for PPII_P around ~ 228 nm, indicating strong PPII propensity as expected. A plot of the estimated free energy of PPII formation versus glycine for each guest residue from Brown et al*.*^[22]^ was plotted against the molar ellipticity measured at 228 nm for each peptide in this study [Fig. S1(b)]. The linear correlation confirms the designed peptides have the expected relative PPII propensities.

**Table I:** The name of each peptide in this study includes the guest residue.

Peptide name	Molecular weight (g/mol)	Peptide sequence	Bulkiness of guest residue (Å^2^)^[23]^	Hydration potential of guest residue (kcal/mol)^[24]^	Propensity of guest residue ΔΔG (kcal mol^−1^)^[22]^
PPII_A	1309.2	Ac-GPP**A**PPGGPP**A**PPGC-NH_2_	11.50	0.3	− 0.21
PPII_I	1393.4	Ac-GPP**I**PPGGPP**I**PPGC-NH_2_	21.40	0.7	0.44
PPII_L	1394.6	Ac-GPP**L**PPGGPP**L**PPGC-NH_2_	21.40	0.5	− 0.44
PPII_N	1395.4	Ac-GPP**N**PPGGPP**N**PPGC-NH_2_	12.82	− 0.5	0.05
PPII_P	1319.2	Ac-GPP**P**PPGGPP**P**PPGC-NH_2_	17.43	− 0.3	− 0.71
PPII_Q	1423.4	Ac-GPP**Q**PPGGPP**Q**PPGC-NH_2_	14.45	− 0.7	-0.08
PPII_T	1370.6	Ac-GPP**T**PPGGPP**T**PPGC-NH_2_	15.77	− 0.2	0.41
PPII_V	1365.4	Ac-GPP**V**PPGGPP**V**PPGC-NH_2_	21.57	0.6	0.41
PPII_Hyp	1393.4	Ac-GPP**Hyp**PPGGPP**Hyp**PPGC-NH_2_	–	–	–

Each peptide has two repeats of the GPPXPPG sequence, along with N-terminal acetylation and C-terminal amination.

A range of guest residues was chosen with varying hydration potential, PPII propensity, and bulkiness.

The guest residue is shown in bold for each peptide.

**Figure 1:**
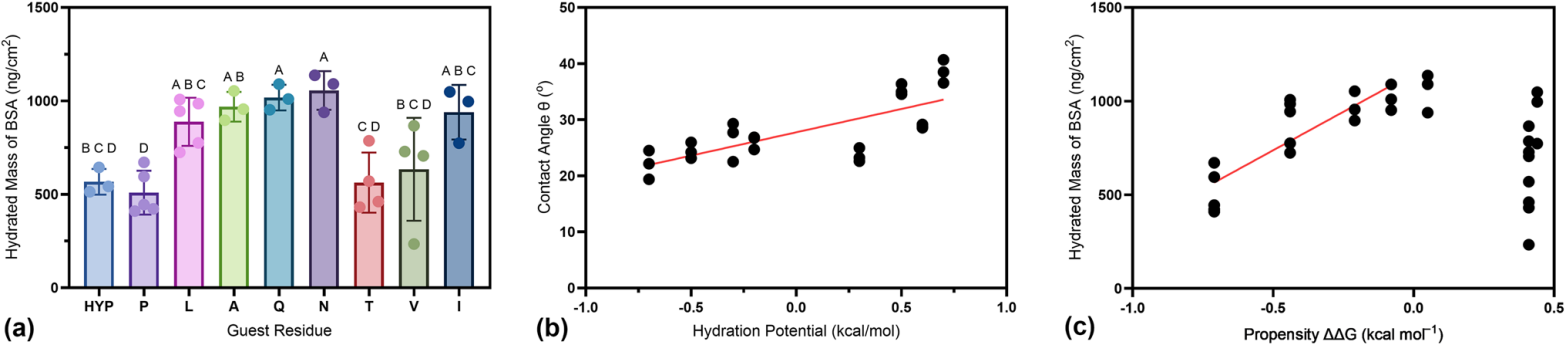
(a) Guest residue impacts the hydrated mass of BSA fouling on monolayers of PPII peptides. Letters represent the results of a Tukey’s post hoc test. Bars that do not share a letter represent data that are statically different. Data are represented by the mean ± standard deviation with *n* = 3–5. (b) Estimated hydration potential for each guest residue from Janin^[24]^ versus measured contact angle of PPII peptide-functionalized gold surfaces. (c) Estimated free energy of PPII formation relative to glycine for each guest residue from Brown et al*.*^[23]^ versus hydrated mass of BSA on PPII peptide-functionalized gold surfaces (note that as estimated free energy of PPII formation relative to glycine increases, PPII propensity decreases). Equations for the linear best fit lines (plotted in red) were obtained from the least squares method [*p*-value = 0.000 for both plots in (b) and (c)]. For (c), linear regression was performed only on peptide samples with negative free energy of PPII formation relative to glycine.

Adsorption experiments were conducted with BSA to generally assess the impact of the guest residue on fouling. First, monolayers of each PPII peptide were adsorbed to a gold surface with adsorption monitored via QCM-D. Figure S2(a) shows an example adsorption profile of peptide for three hours after 10 min of stable baseline. Functionalization was terminated after three hours, followed by a water rinse. Figure S2(b) shows that after three hours of functionalizing peptide to a gold sensor, the adsorbed hydrated mass for all peptides was statistically similar (via single-factor ANOVA), with averages between 310 and 360 ng/cm^2^. BSA was then exposed to the peptide-functionalized surface followed by a final water rinse. Figure 1(a) shows the estimated hydrated mass of BSA that adsorbed on each peptide monolayer. Single-factor ANOVA revealed guest residue to have a significant impact on BSA fouling (*p*-value < 0.05). Peptides containing P as the guest residue had significantly lower fouling than peptides containing L, A, Q, N, and I as the guest residue.

PPII peptide guest residue properties were explored for their correlation with fouling. Bulkiness and hydration potential of guest residues were plotted against the hydrated mass of BSA fouling [Fig. S3(a, b), respectively]. Simple linear regression was conducted and no significant relationship between bulkiness [Fig. S3(a)] or hydration potential [Fig. S3(b)] with fouling was identified. The contact angle of each peptide monolayer sample was measured to assess if the hydration potential scale matched the wettability of the sample. Figure S4 shows the measured contact angles for each prepared peptide monolayer sample, and Fig. 1(b) shows that the contact angle significantly correlated with the hydration potential of the guest residue (*p*-value < 0.05). Figure 1(c) shows that as peptide PPII propensity decreased, fouling increased until the helix was less favorable (note that as the estimated free energy of PPII formation relative to glycine increases, PPII propensity decreases). PPII favorability was determined by our circular dichroism results, where samples with relatively large negative ellipticity at ~ 228 nm, which correlated with a positive free energy of PPII formation relative to glycine, were not included in our regression analysis. PPII propensity in samples with negative free energy of PPII formation relative to glycine was significantly correlated with BSA fouling (*p*-value < 0.05). It was also observed that in samples with positive free energy of PPII formation versus glycine, the error bars appeared to increase compared to peptides with more favorable PPII propensity.

The results in this study suggest that PPII peptides with high proline content have high antifouling properties. In addition, polyproline propensity also appears to be related to antifouling properties for the guest residues in this study. To assess the robustness of this result, we wanted to see if antifouling properties could be maintained with high PPII content (positive band at ~ 228 nm) but higher ability to form hydrogen bonds. Thus, hydroxyproline (Hyp) was selected as a guest residue due to its similar structure to proline, but with the addition of a hydroxyl group. We found that PPII_Hyp showed comparable PPII propensity to PPII_P [Fig. S1(a)], while maintaining low fouling (Fig. 1).

### The relationship between PPII peptide adsorption and BSA fouling

**Figure 2:**
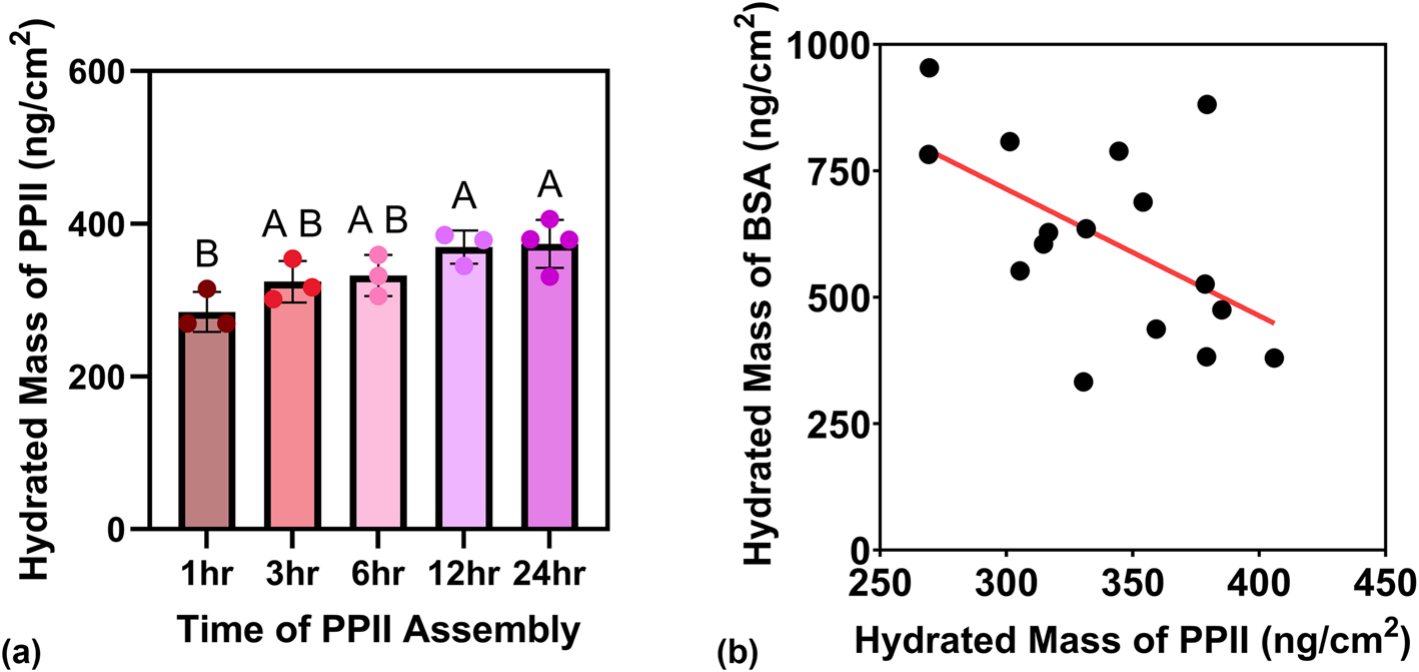
PPII_P loading can be kinetically controlled and significantly impacts BSA fouling. (a) PPII_P hydrated mass loading for 1, 3, 6, 12, and 24 h of exposure times of peptide to the gold-coated QCM-D sensor. (b) PPII_P peptide adsorption and BSA adsorption has an inverse relationship. Letters represent the results of a Tukey’s post hoc test. Bars that do not share a letter represent data that are statistically different. Data are represented by the mean ± standard deviation with *n* = 3–5. The equation of the linear best fit line (plotted in red) was obtained from the least squares method and the linear relationship found to be statistically significant (*p*-value < 0.05).

Previous work has suggested that the high packing density of oligo-prolines increases antifouling properties.^[18]^ In that study, packing density was controlled by length, and only two different peptide lengths (and thus loading amounts) were explored. We aimed to control the packing density directly via exposure time using the same peptide to see if a significant linear correlation between hydrated mass and fouling existed. We chose PPII_P, due to the high antifouling properties observed in Fig. 1. Figure 2(a) shows the hydrated peptide loading after exposure of the gold-coated QCM-D sensor to the peptide solution for 1, 3, 6, 12, and 24 h. As the assembly time increased, the loading significantly increased, as expected from our previous work. ANOVA indicates that assembly time has a significant impact on peptide hydrated mass loading (*p*-value < 0.05). Next, BSA was allowed to adsorb to the samples of varying peptide loading. Figure 2(b) is a plot of the hydrated mass of BSA versus the hydrated mass of peptide. Simple linear regression was performed and a significant correlation between BSA fouling and PPII_P peptide loading was observed (*p*-value < 0.05). These data support that packing density significantly impacts the fouling properties in the PPII peptide system.

### Impact of PPII peptide on hMSC spreading

While previous studies have explored the attachment of mouse fibroblasts to oligo-prolines,^[18]^ no studies have elucidated human mesenchymal stem cell attachment or quantified the spreading of such cells on PPII peptides. hMSCs are promising due to their ability to differentiate into multiple cell types, and controlling cell spreading is crucial for biomaterials design.^[30]^ Thus, hMSCs were seeded onto bare gold, PPII_P, and PPII_L, and allowed to incubate for two hours and their morphology was analyzed. PPII_P and PPII_L were chosen because of their significantly different BSA fouling behavior, observed in Fig. 1.

**Figure 3:**
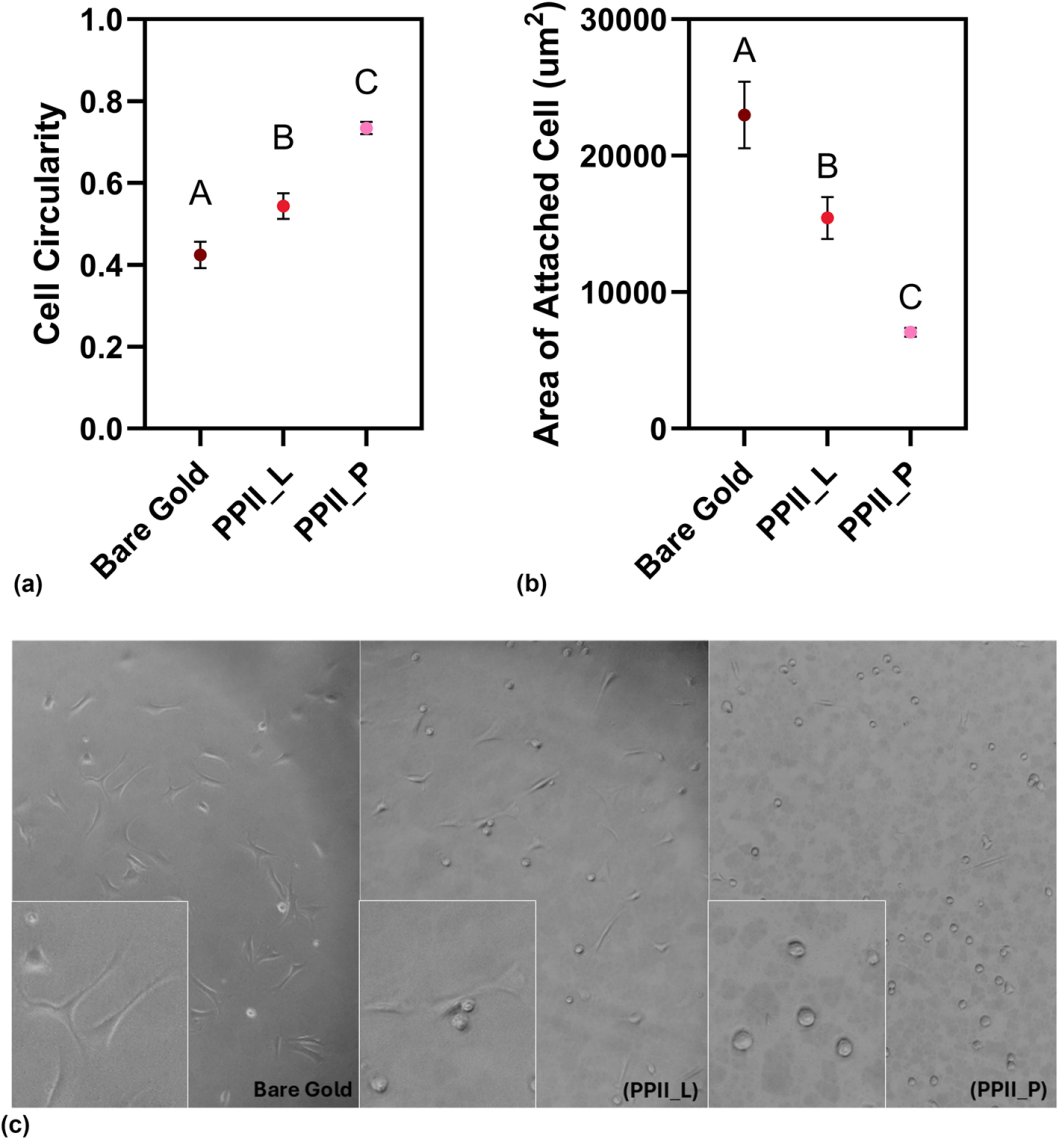
Guest residue impacts hMSC cell spreading on monolayers of PPII peptide. (a) Cell circularity measured after 2 h of hMSC incubation on surfaces with no peptide (bare gold), with adsorbed PPII_L, and with adsorbed PPII_P. (b) Cell area measured after 2 h of hMSC incubation on surfaces with no peptide (bare gold), with adsorbed PPII_L, and with adsorbed PPII_P. (c) Representative pictures of hMSCs on bare gold, PPII_L, and PPII_P after two hours of incubation. Letters represent the results of a Tukey’s post hoc test. Bars that do not share a letter are statistically different. Data are represented by the mean ± 95% confidence interval with *n* = 132–219 cells. Insets show representative cells for each surface.

Figure 3(a) shows the average hMSC cell circularity, where a perfect circle is indicated as a value of one, while a straight line is indicated as a value of zero. Unattached cells generally have a round morphology while attached cells have a more spread and spindle-like morphology. After two hours of incubation, cells cultured on the gold surface with no peptide had a circularity of 0.42 ± 0.19, while cells cultured on PPII_L and PPII_P had a circularity of 0.54 ± 0.19 and 0.73 ± 0.11, respectively (reported as the mean ± standard deviation). ANOVA indicated that the surface had an impact on circularity (*p*-value < 0.05), with all three groups statistically different from each other. Figure 3(b) shows the average hMSC cell area on bare gold, PPII_P, and PPII_L peptide-coated surfaces. ANOVA indicated that the surface treatment has a significant impact on cell area (*p*-value < 0.05), with post hoc testing revealing statistically significant differences between all groups. Bare gold had the highest cell area (23,000 ± 14,000 µm^2^/cell), followed by cells on PPII_L (15,000 ± 9100 µm^2^/cell). Cells on PPII_P had the lowest cell spreading (7100 ± 2400 µm^2^/cell, reported as the mean ± standard deviation). Therefore, while both PPII peptides reduced hMSC cell spreading compared to the bare gold control, PPII_P prevented the most spreading, consistent with the BSA antifouling results shown in Fig. 1. Figure 3(c) shows representative images of the cell spreading, with noticeable differences between the surfaces. Cells incubated on PPII_P are still spherical in shape, while cells on PPII_L are a mixture of spherical and spreading morphologies and cells on the bare gold control are highly spread. These data suggest that the PPII peptide guest residue can be used to modulate hMSC spreading in a two-dimensional cell culture format.

## Conclusions

A PPII peptide guest residue system was designed to adsorb to gold and study the impact of guest residue on BSA fouling and hMSC spreading. Guest residue was found to have a significant effect on BSA fouling, with the proline and hydroxyproline guest residues having high antifouling properties. Guest residue hydration potential was also found to significantly impact the wettability of the peptide-functionalized gold surface. Interestingly, no correlation between hydration potential or side chain bulkiness and BSA adsorption could be identified in this study. However, for the guest residues in this work, there was a significant relationship identified between PPII propensity and BSA fouling. In addition, hydrated mass loading of the PPII_P peptide was shown to be directly correlated with BSA fouling. PPII peptides were found to significantly impact hMSC area and circularity and correlated with BSA adsorption results, where cells on PPII peptides with proline as the guest residue had the lowest cell spreading and highest circularity. Collectively, this research indicates that the presence of proline/hydroxyproline is important in PPII peptide antifouling behavior and implicates PPII propensity as an important factor for further study. In addition, we show that PPII peptide guest residues can be utilized to control the spreading and adhesion of clinically relevant cells such as hMSCs.

## Supplementary Information

Below is the link to the electronic supplementary material.

Supplementary file1 (DOCX 501 KB)

## Data Availability

Data are available upon reasonable request.
