# Supplementary material for: Impact of coverage and guest residue on polyproline II helix peptide antifouling
Source: MRS Commun. 2024 Nov 11;14(6):1134–41. doi: 10.1557/s43579-024-00674-w (PMC11618189; doi:10.1557/s43579-024-00674-w)
Supplement: Supplementary file 1 — Supplementary file1 (DOCX 501 KB) [file 43579_2024_674_MOESM1_ESM.docx]

**Supplemental Information**


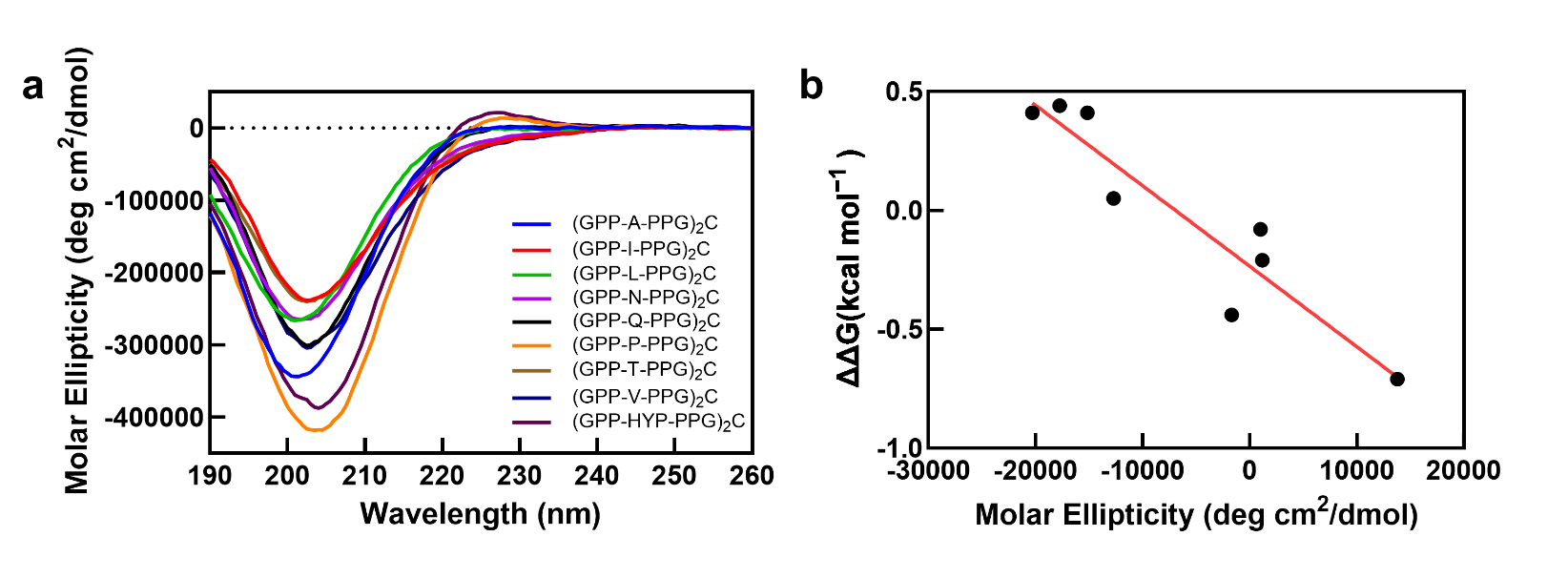


**Figure S1.** (a) CD spectra of 100 µM samples of PPII peptide. (b) Estimated free energy of PPII formation relative to glycine from Brown *et al^22^* for each guest residue versus molar ellipticity measured at 228 nm for each peptide in this study. The equation of the linear best fit line (plotted in red) was obtained from the least squares method (p-value = 0.001).

**
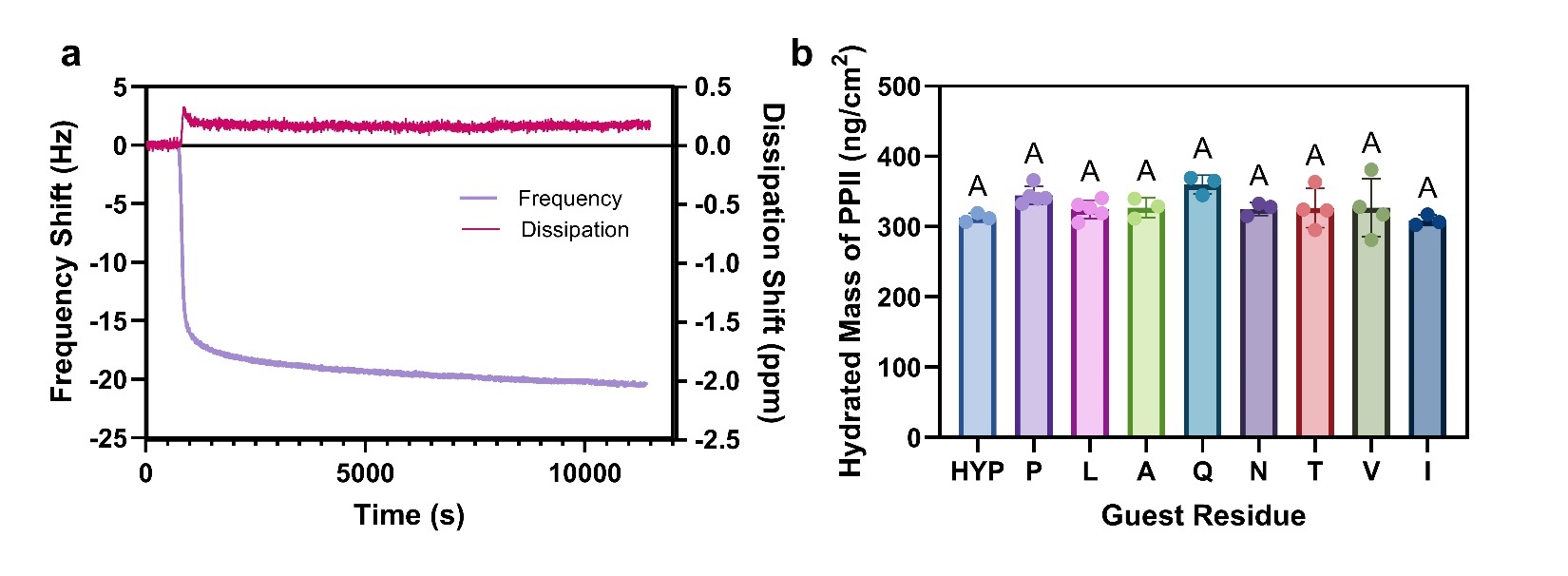
**

**Figure S2.** (a) Example peptide adsorption with frequency and dissipation shifts measured using QCM-D. (b) Estimated mass for each peptide (signified by guest residue) after adsorption for three hours via QCM-D. Single factor ANOVA was conducted on mass data, and no evidence was found to suggest guest residue has a significant impact on the total mass of adsorbed peptide. Data are represented by the mean ± standard deviation with n = 3-5.


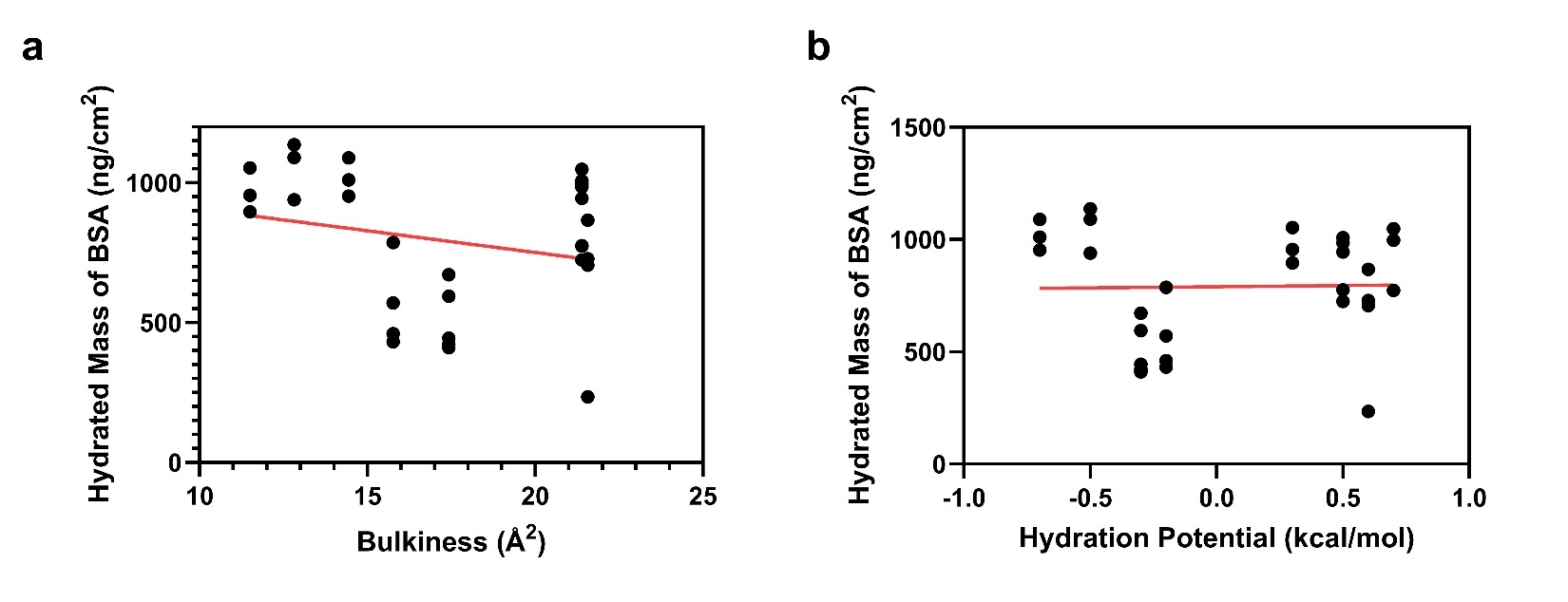


**Figure S3.** (a) Estimated bulkiness for each guest residue from Zimmerman *et al.*^23^ versus hydrated mass of BSA on monolayers of PPII peptides. (b) Estimated hydration potential for each guest residue from Janin.^24^ versus hydrated mass of BSA on monolayers of PPII peptides. The equations for linear best fit lines (plotted in red) were obtained from the least squares method, and the linear relationships were not significant (p-value = 0.216 and p-value = 0.916 for a and b, respectively).

**
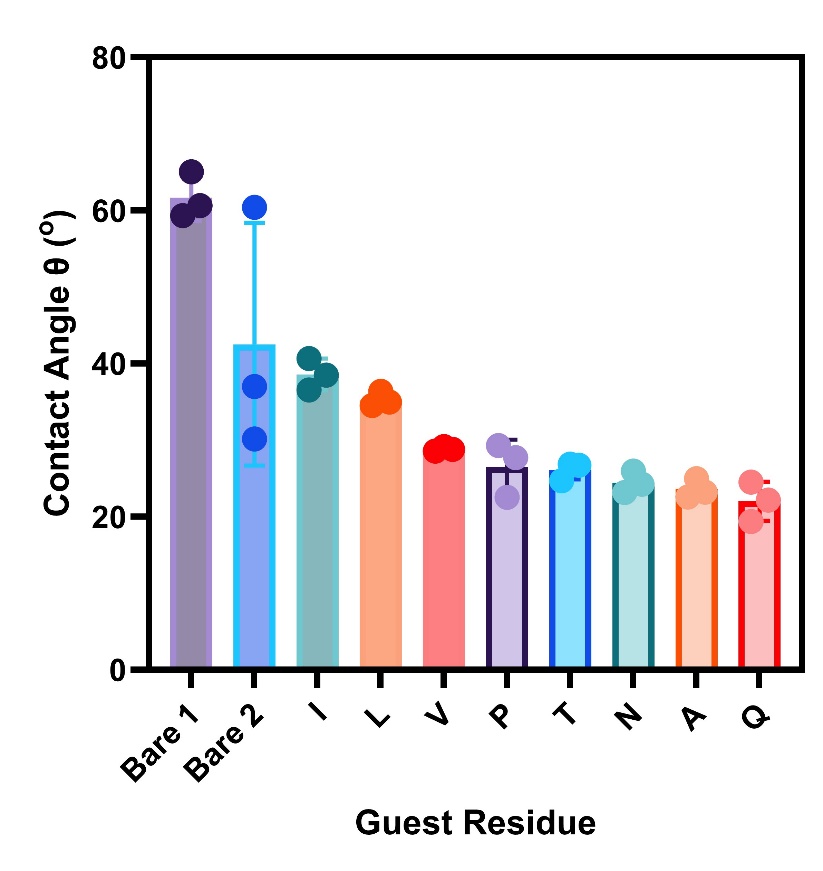
**

**Figure S4.** Measured contact angle of PPII peptides with different guest residues, arranged in decreasing order. Bare 1 refers to a bare gold control that was not soaked in water prior to measuring contact angle. Bare 2 refers to a bare gold control that was soaked in Millipore-filtered DI water for a similar amount of time as the peptide samples.
